# Infaunal macrobenthic community dynamics in a manipulated hyperhaline ecosystem: a long-term study

**DOI:** 10.1186/2046-9063-9-20

**Published:** 2013-11-06

**Authors:** Bruno Bellisario, Claudio Carere, Fulvio Cerfolli, Dario Angeletti, Giuseppe Nascetti, Roberta Cimmaruta

**Affiliations:** 1 Department of Ecological and Biological Sciences, Ichthyogenic Experimental Marine Centre (CISMAR), Tuscia University, Borgo Le Saline, 01016 Tarquinia, VT, Italy

**Keywords:** Hyperhaline habitat, Wetlands, Central Tyrrhenian Sea, Biomonitoring, Macroinvertebrates community, Long-term study, Species specialization index, Biodiversity homogenization

## Abstract

**Background:**

Understanding the responses of ecological communities to human-induced perturbations is crucial for establishing conservation goals. Ecological communities are dynamic entities undergoing fluctuations due to their intrinsic characteristics as well as anthropogenic pressures varying over time. In this respect, long-term studies, based on large spatial and temporal datasets, may provide useful information in understanding patterns and processes influencing the communities’ structure. Theoretical evidence suggests that a role of biodiversity is acting as a compensatory buffer against environmental variability by decreasing the temporal variance in ecosystem functioning and by raising the level of community response to perturbations through the selection of better performing species. Therefore, the spatial and temporal changes in the specialization of the community components may be used as an effective tool to monitor the effects of natural and anthropogenic alterations of the environment in dynamic systems. We examined the temporal dynamics of macroinvertebrate community structure in the hyperhaline habitat of Tarquinia Saltworks (central Italy). We aimed at: (i) investigating the relationships between the level of community specialization and the alterations of the environment across fourteen years; (ii) comparing the ability of aggregate community parameters such as the average abundance vs. species specialization in describing patterns of community composition.

**Results:**

We arranged the data in three sub-sets according to three periods, each characterized by different environmental conditions. The mean abundance of sampled macroinvertebrates showed a significant change (*p* < 0.01) only in the community inhabiting the saltwork basin closely connected to the sea, characterized by the highest environmental variation (i.e. the coefficient of variation, *CV*, of the aggregate environmental variability over the study period, *CV*_range_ = 0.010 - 0.2). Here we found marine species like *Modiolus adriaticus* (Lamarck, 1819), *Neanthes irrorata* (Malmgren, 1867), and *Amphiglena mediterranea* (Leydig, 1851), which inhabited the saltworks during the halt period but disappeared during the subsequent eutrophication phase. Conversely, species specialization showed a significant decrease for each sampled community in the presence of habitat degradation and a recovery after ecological restoration. The widest fluctuations of specialization were recorded for the community inhabiting the saltwork basin with the highest long-term environmental variability.

**Conclusions:**

Recent advances have shown how the increased temporal and spatial variability of species’ abundance within the communities may be a signal of habitat disturbance, even in the absence of an apparent decline. Such approach could also be used as a sensitive monitoring tool, able to detect whether or not communities are subjected to increasing biotic homogenization. Also, the increased functional similarity triggered by habitat degradation may impact on species at higher trophic levels, such as the waterbirds wintering in the area or using it as a stopover during migration.

## Background

The dynamic responses of the species to both environmental fluctuations and interspecific interactions may exert a strong influence on the structural assemblages of communities [1,2]. Theory suggests that not only the magnitude, but also the temporal frequency of the environmental fluctuations matter in altering the structural and functional composition of ecological communities (e.g. coarse- vs. fine-grained environmental changes *sensu* Levins [3]). For instance, the frequency of the occurrence of the environmental variations may drive the overall resource allocation within the communities, thereby, influencing the ecological specialization of the species and populations. When the environmental fluctuations are small and temporally spaced, communities should be composed of species locally adapted to the relatively stable environment, even if the environmental conditions are severe [3]. Conversely, marked and frequent environmental fluctuations should promote the interchange of different arrays of species with a high diversity of functional traits undergoing a temporal turn-over according to changing conditions [4].

Monitoring the changes in community structure may help detect early signals of environmental disturbance. In particular, a number of studies highlight a link between environmental fluctuations, including anthropogenic disturbance, and biodiversity loss [5,6]. Ecologists have been long interested in the loss of biodiversity associated with environmental changes focusing mainly on the taxonomic diversity of communities. However, the functional diversity (i.e., the variation of species functional traits within a community [7,8]), is a primary aspect of biodiversity known to be an accurate predictor of ecosystem functioning [9-11]. There is also growing evidence that both functional and taxonomic diversity are linked to shifts of ecosystem processes [12].

A crucial issue is the trade-off between specialist and generalist species in explaining the functioning of key ecosystem processes [13,14], as specialist species should be more affected than generalists by environmental changes because of the strong association with their particular niche [15]. Indeed, the concept of specialization is closely related to the prediction of adaptive responses of species in heterogeneous and/or fluctuating environments [3], and its definition relies on "one of the most confusing, and yet important topics in ecology," the niche concept [16]. The degree of specialization is now considered as an informative component of community structure [17]. Therefore, the use of appropriate metrics able to detect spatio-temporal changes in the specialization of the community components is essential to evaluate the effects of both natural and anthropogenic alterations of the environmental conditions in dynamic systems [18]. In particular, a correct distinction between different facets of ecological specialization is required to understand the effects of habitat changes on the biotic homogenization, which can reshuffle existing species distributions by replacing local-adapted species with more widespread and generalist ones, reducing the spatial diversity of communities [19]. In other words, if the alteration of the environment acts as a non-random filter by selecting the species with a higher fitness in the modified ecosystem [20], then the biotic homogenization influences the replacement of 'losers’ species by 'winners’, which increases the spatial similarity of species’ functional traits over time [21]. As a consequence, impacted communities should have lower levels of specialization, since generalist species may better tolerate the environmental changes associated with disturbance (i.e., loss of habitats, hence niches [22]).

Coastal aquatic ecosystems are extremely dynamic habitats where the environmental variations occur over small temporal and spatial scales [23]. In particular in saline systems, this variability is related to inundation/evaporation cycles, which generate highly fluctuating conditions in terms of both frequency and magnitude of changes in the environmental parameters. These fluctuations produce an enduring state of elevated disturbance on the local macroinvertebrate communities [23], which are then subject to large spatial and temporal variation in abundance and diversity. These habitats are, therefore, particularly suitable to implement the use of functional based metrics, which should reveal the effects of environmental changes on the community structure. Such an approach may help capture effects otherwise masked by aggregate community properties [14,24-26]. For example, to date, there are many studies linking environmental fluctuations to the abundance and species diversity of macrobenthic communities from coastal lagoons [27,28], but just a few studies analyzed their impact on the species specialization and functional diversity [22,29,30].

In this study, we examined long-term changes on the macroinvertebrate community composition of a hyperhaline habitat represented by halted saltworks. The aim was to test whether and how the level of community specialization could be impacted by both natural fluctuations and human-induced alterations in the environment. About fourteen years of monitoring activities have been divided into three main temporal blocks centered on a three-year period of management activities aimed at restoring the water circulation between the saltwork basins. The variation of aggregate community properties (i.e. average abundance) and species specialization (i.e. temporal variation of abundance) were calculated for each array of environmental conditions to check for the reliability of such metrics in describing patterns of community structure. Also, the trends of these metrics were analyzed in basins characterized by different environmental variability to verify their sensitivity in comparing coarse- vs. fine-grained environmental changes.

## Results

**Figure 1:**
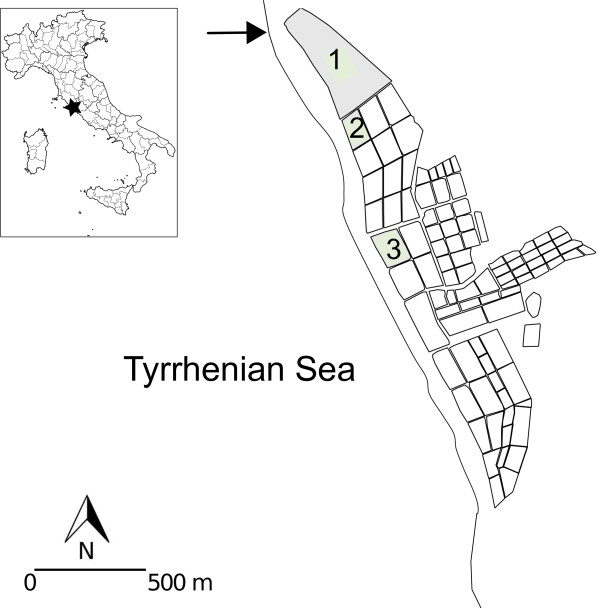
**The study area with the sampling sites (in grey).** The arrow indicates the channel for marine water refill.

**Figure 2:**
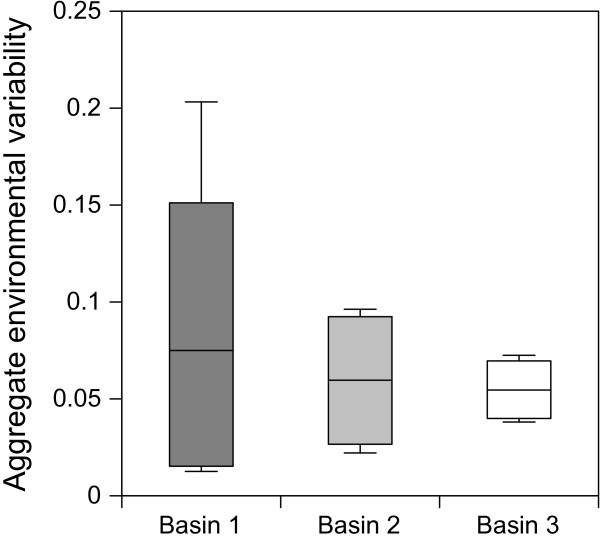
**Degree of environmental variability expressed as aggregate coefficients of variation of the sampled physical-chemical parameters in different sampling sites.** Boxplots show the cumulative coefficient of variation (SD/mean) of salinity, pH, dissolved oxygen concentration, and temperature during the entire study period (1997-2010). Dark grey box is for basin 1, light grey box for basin 2, and white box for basin 3.

**Figure 3:**
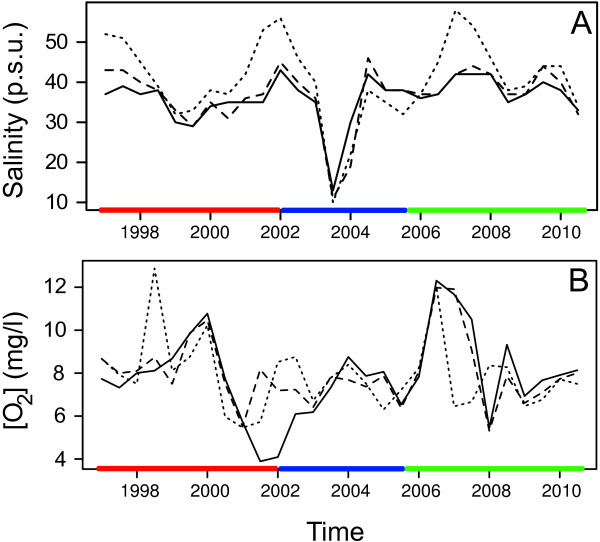
**Temporal trend of salinity (A) and dissolved oxygen concentration (B).** Solid line is for basin 1, dashed line for basin 2, and dotted line for basin 3. Red, blue, and green lines on the bottom of the graphs represent halt (1997-2002), eutrophication (2003-2005), and post-recovery (2006-2010) periods, respectively.

The degree of environmental variability, expressed by the aggregate coefficient of variations of the measured parameters within the sampled communities (Figure 1), decreased following a spatial trend from basin 1 to 3 (Figure 2). Salinity and dissolved oxygen concentration accounted for most of the variation in all sampled basins, as indicated by the first two axes of the PCA (Table 1), which explained more than 90% of the total variance. The first principal component alone explained 72% of the total variance mainly attributable to the salinity (*r* = 0.96), followed by the dissolved oxygen concentration (*r* = -0.24). Both parameters showed marked fluctuations (Figure 3) observing an abrupt decline of both salinity and dissolved oxygen concentration during the eutrophication period followed by a slight increase during the post-recovery (Figure 3).

**Table 1:** Percentage of variance explained by the first two axes of the principal component analysis (PCA) and component loadings for environmental parameters

	**PC1**	**PC2**
% variance	71.996	27.468
Salinity (p.s.u.)	0.9618	0.2288
O_2_ (mg/l)	-0.2402	0.9686
T (°C)	0.131	0.09686
pH	-0.00611	0.007522

PCA was performed on the coefficient of variation (SD/mean) of each parameter measured for each period (Halt = 1997-2002, Eutrophication = 2003-2005, Post-recovery = 2006-2010).

The benthic macroinvertebrates recovered are listed in Table 2, with the data aggregated per study period. Twenty taxa were identified at the species level and ten at the genus to family level. The species richness (*S*) dropped from 21 taxa during the halt period to 14 during the eutrophication phase and then recovered up to 24 taxa during the post-recovery.

**Table 2:** List of the benthic macroinvertebrates found in the Tarquinia Saltworks during the fourteen years of the study

						**Pre-recovery**		**Post-recovery**
***N***	**Phylum**	**Class**	**Order**	**Taxon**	**Habitat**	**Halt**	**Eutrophication**	
1	*Mollusca*	Gastropoda	Littorinimorpha	*Hydrobia acuta* (Draparnaud, 1805)	Brackish	x	x	x
2			Caenogastropoda	*Cerithium vulgatum* (Bruguière, 1792)	Marine	x	x	x
3			Neogastropoda	*Nassarius corniculum* (Olivi, 1792)	Brackish/Marine	x		x
4			Pulmonata	*Ovatella myosotis* (Draparnaud, 1801)	Saltmarsh	x	x	
5			Cephalaspidae	*Haminoea* sp. (Turton & Kingston in Carrington, 1830)	Marine			x
6		Bivalvia	Veneroida	*Abra segmentum* (Récluz, 1843)	Brackish	x	x	x
7				*Cerastoderma glaucum* (Bruguière, 1789)	Marine/Saltworks	x	x	x
8			Mytiloida	*Mytilaster* sp. (Monterosato, 1884)	Marine	x		x
9				*Modiolus adriaticus* (Lamarck, 1819)	Marine	x		
10	*Annelida*	Polychaeta	Nainereis (Genus)	*Nainereis laevigata* (Grube, 1855)	Marine	x	x	x
11			Spionida	*Spio decorates* (Bobretzky, 1870)	Marine	x	x	x
12			Capitellidae (Family)	*Capitella capitata* (Fabricius, 1780)	Cosmopolitan	x	x	x
13			Phyllodocida	*Neanthes irrorata* (Malmgren, 1867)	Marine	x		
14				*Perinereis cultrifera* (Grube, 1840)	Marine	x	x	x
15				*Ophiodromus pallidus* (Claparède, 1864)	Marine			x
16			Sabellida	*Amphiglena mediterranea* (Leydig, 1851)	Marine	x		
17			Orbiniidae (Family)	*Protoaricia oerstedi* (Claparède, 1864)	Marine			x
18		Clitellata	Oligochaeta	Incertae sedis	Cosmopolitan	x	x	x
19	*Arthropoda*	Malacostraca	Isopoda	*Idotea balthica* (Pallas, 1772)	Subtidal	x	x	x
20				*Sphaeroma serratum* (Fabricius, 1787)	Marine	x		
21			Amphipoda	*Monocorophium insidiosum* (Crawford, 1937)	Marine	x	x	x
22				*Gammarus aequicauda* (Martynov, 1931)	Marine	x	x	x
23				*Ericthonius sp.* (Milne-Edwards, 1830)	Marine			x
24				*Microdeutopus spp* (Costa, 1853)	Brackish/Marine			x
25		Insecta	Diptera	*Chironomus* sp (larvae) (Meigen, 1803)	Cosmopolitan	x	x	x
26				(Others) (larvae)	Cosmopolitan	x		x
27	*Nemertea*	Enopla	Monostilifera	Ototyphlonemertidae? (Diesing, 1863)	Intertidal			x
28	*Platyhelminthes*	Rhabditophora	Polycladida	*Stylochus* sp. (Ehrenberg, 1831)	Marine			x
29	*Cnidaria*	Anthozoa	Actiniaria	Actiniidae, (Family) (Rafinesque, 1815)	Marine			x
				*S*		21	14	24

*N* is the number of sampled taxa and *S* the total number of macroinvertebrates per period. Two samplings per year were carried out, during winter and summer, and the data were cumulated within each time period (Halt = 1997-2002, Eutrophication = 2003-2005, Post-recovery = 2006-2010).

**Figure 4:**
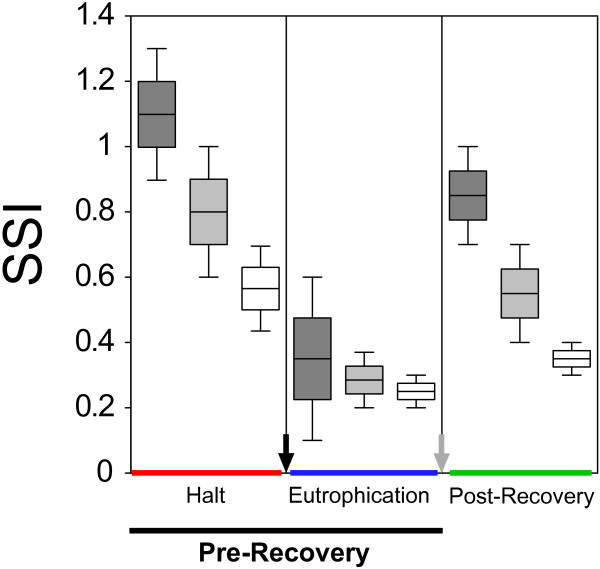
**Boxplots showing the distribution of SSI (Species Specialization Index) in different periods (Halt, Eutrophication, Post-recovery).** Dark grey boxes are for basin 1, light grey boxes for basin 2, and white boxes for basin 3. The black arrow indicates the first algal bloom and grey arrow the start of recovery actions.

A preliminary analysis showed a significant variation of both the abundance (Kruskall-Wallis one-way ANOVA *H* = 17.85, *p* < 0.01) and the degree of specialization (SSI) (Kruskall-Wallis one-way ANOVA *H* = 5.653, *p* = 0.05) of sampled macroinvertebrates in basin 1 across the entire study period. However, the communities in basins 2 and 3 did not show any significant variation of abundance (Kruskall-Wallis one-way ANOVA *H* < 0.3, *p* > 0.80 in both cases), although SSI significantly varied in both communities (Kruskall-Wallis one-way ANOVA *H* > 9 and *p* < 0.01 in both cases). The distribution of SSI across the three periods followed the same trend in all sampled communities (Figure 4) with a drastic depletion at the beginning of the first algal bloom followed by an increase during the post-recovery period. The average SSI decreased spatially from basin 1 to basin 3 during both the halt and the post-recovery period (Figure 4) following the north to south axis of the area. During the eutrophication period, SSI showed quite similar values in all three basins.

**Figure 5:**
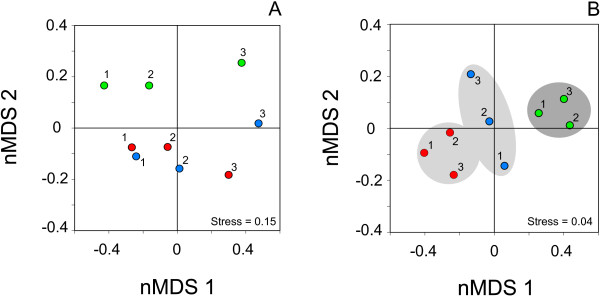
**Non-metric multidimensional scaling (nMDS) plots showing the ordination patterns of macroinvertebrate communities. A** is the nMDS plot based on the log-transformed abundance of the species and **B** is the same plot based on the species specialization index (SSI). Red, blue, and green circles represent halt (1997-2002), eutrophication (2003-2005), and post-recovery (2006-2010) periods, respectively. Distances between points represent the difference according to the Bray-Curtis dissimilarity, with light grey and dark grey ellipses showing the pre- and post-recovery clustering of points, respectively.

The communities did not show any significant difference across periods when considering the abundance of macroinvertebrates (PERMANOVA *F* = 0.905, *df* = 2, *p* = 0.511). This was consistent with the pattern of points separation in the nMDS plot (Figure 5A), and is lacking a clear separation of communities across different sampling sequences. Conversely, the degree of species specialization captured the amount of variation in community structure (PERMANOVA *F* = 2.999, *df* = 2, *p* < 0.01) as observed by the pattern of points separation in the nMDS ordination plot (Figure 5B). The data showed a well-defined pattern of association with communities subdivided in two main groups following a 'time-sequence’ gradient from pre- to post-recovery activities.

## Discussion

We investigated how the temporal variability of the environmental features of a hyperhaline system could influence the responses of macroinvertebrates in their use of habitat resources, i.e. their degree of specialization. In particular, we compared the resolution power of the widely investigated aggregate community properties, such as the average abundance, against the variation of the parameter itself (i.e. the variation of abundance) by considering this latter as a proxy of the degree of specialization [18,31,32].

Most of the environmental variability within the study area was due to the spatial and temporal fluctuations in the level of salinity, which is confirmed as the main driving force in the structuring process of both benthic communities and species genetic structuring in the study site [33,34]. This finding is in agreement with the bulk of literature showing that abiotic processes (e.g. salinization) exert a direct impact on communities’ composition [35] or indirect effects that may influence predatory and/or competitive interactions, which enhances top–down effects on species occurrence, abundance, and biomass [36]. Notably, some of the sampled communities did not show any significant pattern of variation in abundance along the main environmental gradient represented by salinity. Macroinvertebrate communities whose structure did not show any substantial temporal change in abundance inhabited the saltwork basins 2 and 3. These basins are less influenced by marine waters compared to basin 1 and are subjected to environmental conditions that were the less variable across the three periods considered and were always the most extreme. On the contrary, the macroinvertebrate community inhabiting basin 1 showed a significant variation in abundance associated with a high variation of physical-chemical parameters both before and after the restoration action (Figure 2).

However, an approach simply relying on abundance may fail in describing spatial and temporal variability of communities, as their composition may be altered without detectable changes in aggregate community properties [37,38]. Measuring the amplitude of the variation in abundance, density, or biomass may capture effects otherwise not detected when measuring the traits themselves [25,26]. Evaluating the variability of the descriptors of macrobenthic communities can, therefore, provide insights into the structuring processes of the community itself, but this goal requires assessments that are carried out over multiple temporal and/or spatial scales [39,40]. This multiple approach has been reliable in quantifying the degree of specialization of species by overcoming density-dependent biases and being statistically independent of the average species density [18]. Even in the absence of an apparent decline of abundance or biomass, an increased temporal and spatial variability of such traits may be the signal of anthropogenic disturbances (e.g. fishing overexploitation [39] and legacies of historical agriculture [40,41]). A conspicuous loss of functional specialization may also be associated with an increase in species richness, as shown by a long-term study on the fish communities of a coastal lagoon undergoing marked environmental degradation [22]. This paradox was generated since the decreasing species were the most specialized and strictly associated with the most degraded habitats, while the newly found species, responsible for the increase in richness, were functionally redundant with those already present in the community [22].

Our findings well fall within this framework even if we recorded steady values of abundance and only a slight increase in richness with 24 taxa sampled during the post-recovery against 21 from the halt period. We found that assessing the variation of the degree of species specialization highlights significant changes within the sampled communities and puts temporal and spatial variability of these traits in relation with environmental impacts. Indeed, our longitudinal study across fourteen years includes abrupt changes (halt, eutrophication, and post-recovery of the saltworks), which clearly distinguish periods characterized by different environmental conditions and disturbance [42]. In particular, the halt period caused a progressive habitat degradation culminating in the eutrophic period and followed by the works of environmental recovery with a strong impact on the saline system. Accordingly, SSI decreased through the halt period, reached its minimum during the eutrophication period, and then significantly recovered after the restoration. The same trend of SSI was recorded in all three communities analyzed (i.e. saltwork basins), thus efficaciously reflecting the effects of environmental changes (Figures 4 and 5B). On the contrary, the average abundances of sampled macroinvertebrates failed to detect this change in two out of three basins (n. 2 and 3) characterized by more steady assets of physical-chemical parameters (Figure 5A).

Temporal changes in the degree of specialization were associated with the environmental fluctuations, which were in turn related to the different states of the habitat. The highest values of SSI have been recorded during the halt period, when the lack of human intervention modified the environmental conditions allowing marine species to enter and colonize the saltworks. Accordingly, basin 1 was the one with the highest values of SSI. This basin is closely connected to the sea and we found marine species like *Modiolus adriaticus* (Lamarck, 1819), *Neanthes irrorata* (Malmgren, 1867), and *Amphiglena mediterranea* (Leydig, 1851) that inhabited the saltworks during the halt period but disappeared during the subsequent eutrophication phase (Table 2). However, when the levels of habitat degradation became so high to cause eutrophication and algal blooms, SSI values dropped to a minimum with similar values in all three of the basins until the recovery work was carried out. The post-recovery monitoring showed quite high values of SSI, indicating a re-establishment of the communities after the perturbation caused by the recovery actions, which, however, did not reach the values observed during the closure period even if the overall number of taxa recorded was the highest (Table 2). This is likely due to the peculiar features of the saltworks habitat during the closure, when the lack of maintenance made the environment suitable for a number of species (as the already mentioned *M. adriaticus*, *N. irrorata,* and *A. mediterranea*) unfit for either the eutrophic or managed (i.e., restored) habitat.

Therefore, our results underline that maintaining a remarkable level of spatial and temporal heterogeneity is crucial for guaranteeing a high biodiversity at the community level. Indeed, the replacement of specialists by more generalist species may have severe consequences on both community and ecosystem functioning (i.e. the so-called functional homogenization of biodiversity) and decrease the synchronization and variability in the responses to disturbance between connected communities [19].

## Conclusions

As coastal aquatic environments are among the most dynamic and impacted ecosystems, it is crucial to understand how the spatial and temporal variability of the environmental conditions may alter the biological composition of their animal communities. Here, we showed how changes in the frequency and magnitude of environmental fluctuations relate to the degree of temporal specialization of macroinvertebrate species in spatially defined communities. Our results support recent advances in the biotic homogenization of biodiversity [19], which relies on the measurement of the increase in similarity of species’ functional traits over time. Measurements based on the species’ contribution to the specific characteristics of community specialization (e.g. variation of density, abundance, or biomass) are considered promising tools in detecting impacts of climate or human-induced alterations on communities [17]. Simple properties of community assembly, i.e. the average abundance of sampled species, failed to detect changes within and between communities facing environmental fluctuations, while the species specialization index (SSI) significantly varied throughout the sampling periods within the macroinvertebrate community.

Long term studies linking environmental changes and impacts to the marine community structure are now emerging [43-45], while in hyperhaline habitats data are still quite scanty (but see [46]) making this study the first case assessing the relationships between the level of community specialization and the long term alteration of a hyperhaline environment.

These results have conservation implications, since an increased replacement of specialists by more generalist species may be the signal of a deterioration of environmental conditions, which reduces the spatial and temporal variability of communities facing disturbance. This increased similarity may have effects on species at higher trophic levels, like the avifauna, by reducing trophic niches and by increasing extirpation rates via intensified species–specific interactions (e.g., functionally similar species might utilize the same spatial resources). Coastal wetlands need constant monitoring and management based on a comprehensive ecosystem approach that links the correct management of the physical-chemical characteristics for the maintenance of community specialization up to food web integrity.

## Materials and methods

### Study area

The study area is a hyperhaline coastal habitat, the Tarquinia Saltworks, located along the Tyrrhenian coasts of northern Latium in central Italy (42°12’ N, 11°43’ E). Despite the saltworks being an artificial ecosystem, they are recognised as wetland areas according to the Ramsar Convention Bureau [47]. Nearly 100 shallow basins compose the Tarquinia Saltworks, which are connected either directly or by a system of drainage channels and fed by the marine water entering a main channel located north of the area (Figure 1).

The long term ecological monitoring of the Tarquinia Saltworks provided a unique opportunity for testing the influence of recent important environmental changes on the local resident communities and species. The salt production was halted in 1997 with a consequent reduction of the water flow and an increase in organic and inorganic matter sedimentation resulting in several episodes of eutrophication starting with an algal bloom in 2003 [42]. During 2005-2006, recovery actions were carried out in the frame of a LIFE project aimed at restoring the water flow and basin depth (LIFE02NAT/IT/8523; http://www.unitus.it/life). The habitat was heavily impacted by this action involving bottom handling and high water flow for about one year. During the subsequent years, the lack of maintenance progressively started to drive the system toward a new state of alteration in the hydrological and trophic conditions.

We analyzed three sampling sites where long-term information about the main chemical–physical parameters and the abundance of macroinvertebrates have been recorded. Benthic communities in each basin were sampled twice a year (a mid-winter and a July-summer sampling) over fourteen years (1997-2010) for an overall number of 28 samples. At the same time the values of salinity, dissolved oxygen concentration, pH, and temperature were recorded. Quantitative sampling was carried out with a Van Veen Grab (0.06 m^2^ and 8 cm depth) and sieved with a 0.5 mm mesh size sieve. The samples were stored in formalin solution (4% formalin + 96% sample/seawater) to preserve their integrity for subsequent *ex-situ* analyses, which involved filtering them on a 1 mm mesh-size funnel-shaped sieve. Sorted macroinvertebrates were then identified to the lowest taxonomic level using a dissection microscope. Further details on the sampling strategy can be found elsewhere [34,42].

The fourteen years of the study were divided in three main periods: the first one follows the halt of the saltworks and was characterized by a progressive deterioration of the habitat conditions (halt, 1997-2002); the second period was characterized by eutrophication and started in 2003 in coincidence with the first algal bloom (eutrophication, 2003-2005); the third period started immediately after the recovery works of the LIFE02NAT/IT/8523 (post-recovery, 2006-2010). We defined the halt and eutrophication periods as the pre-recovery period.

### Assessment of environmental variability

The hydrological isolation/connectivity between the basins within the study area gave rise to a patchy geographic pattern in the variability of the environmental parameters, in particular, salinity and dissolved oxygen [33], which contributes to a marked species turnover and a strong temporal variability in community composition [34]. We quantified the degree of environmental variability by considering the coefficient of variation of the main physical-chemical parameters measured within each period. The coefficients of variation were subject to a principal component analysis (PCA) to reduce the variation in the original dataset into a single component describing the environmental variability within the sampling sites.

### Assessment of specialization

Although ecological specialization is one of the main concepts in ecology and conservation, the numerous existing definitions and metrics require an explicit statement for applied purposes [48]. The degree of specialization of a species should be ideally measured by considering the width of the ecological niche in all its dimensions (e.g. the *n*-dimensional hypervolume, *sensu* Hutchinson [49]). This implies that the quantification of specialization is highly context-dependent and relies on the type of organism studied, the spatial/temporal scale of investigation [48], and the ecological mechanisms involved (see Poisot et al. [15] for an extensive review on different mechanisms driving the evolution of ecological specialization). However, a simple yet sensitive measure of specialization can be derived by the position and shape of species response in abundance, density, or biomass to a given 'resource’ gradient [18,31,32]. Accordingly, we considered the different habitat conditions as the 'resource’ gradient under which the structuring process of a community occurs, and we quantified the degree of specialization of a species in each community through the Species Specialization Index (SSI). The SSI is the variance of average abundances across the three periods considered, measured by the coefficient of variation (SD/mean) to obtain a metric statistically independent of the average species abundance [18]. Therefore, species with more variable abundance over time should be considered more specialized in habitat use than species with more constant abundance. To avoid an overestimation of SSI, those species recorded only once during the study period were discharged from the analysis.

### Community-wide analysis

We used permutational multivariate analysis of variance (PERMANOVA), with the first axis of PCA (see above) as fixed factor, to test for differences in the composition of macroinvertebrate communities along the environmental gradient throughout temporal sequences. PERMANOVA is a semi-parametric group difference test analogous to multivariate analysis of variance, but with pseudo-*F* ratios and *p*-values generated by permuting the resemblance measures of actual data. Therefore, it is less sensitive to assumptions of parametric tests that are frequently violated by community data sets [50].

We used the Bray-Curtis coefficient to construct resemblance matrices based on the abundance of sampled macroinvertebrates (log-transformed to improve normality) and the calculated specialization index. The *p*-value of significance was tested by performing 999 permutations across separated sets of data (i.e. abundance and specialization) within each group (i.e. time periods). We used the function 'adonis’ , implemented in the R package 'vegan’ [51] for partitioning distance matrices among sources of variation. Although similar to the classic PERMANOVA, the function 'adonis’ is more robust as it can accept both categorical and continuous variables.

Non-metric multidimensional scaling (nMDS) was used to highlight spatial and temporal patterns of community structure based on both the log-transformed abundances and specialization of sampled macroinvertebrates. A stress value ranging from 0 to 1.0 was used to measure the reliability of the ordination with zero indicating a perfect fit and all rank orders correctly represented by the relative distance between all pairs of points in the graph and with values > 0.3 indicating an arbitrary placement of the points in the graph [52].

## Competing interests

The authors declare that they have no competing interests.

## Authors’ contributions

All the authors contributed to this long-term study and were involved at different times. RC, GN, and DA have been working on the environmental monitoring and ecological recovery of the Tarquinia saltworks since 1997. RC, BB, and GN contributed to the conceptual development of the work. BB and RC drafted the manuscript and BB performed the statistical analyses with CC and FC. DA and FC checked and analyzed the physical-chemical data and the macroinvertebrate list. DA, FC, GN, and CC revised the drafted manuscript. All the authors read and approved the final version of the manuscript.
